# Evaluation of DNA Binding, Cleavage, and Cytotoxic Activity of Cu(II), Co(II), and Ni(II) Schiff Base Complexes of 1-Phenylindoline-2,3-dione with Isonicotinohydrazide

**DOI:** 10.1155/2014/215392

**Published:** 2014-03-12

**Authors:** Ramadoss Gomathi, Andy Ramu, Athiappan Murugan

**Affiliations:** ^1^Department of Inorganic Chemistry, School of Chemistry, Madurai Kamaraj University, Madurai, Tamil Nadu 625 021, India; ^2^Department of Microbiology, Periyar University, Salem, Tamil Nadu 636011, India

## Abstract

One new series of Cu(II), Co(II), and Ni(II) Schiff base complexes was prepared through the condensation reaction between 1-phenylindoline-2,3-dione with isonicotinohydrazide followed by metalation, respectively. The Schiff base ligand(L), *(E)*-N′-(2-oxo-1-phenylindolin-3-lidene)isonicotinohydrazide, and its complexes were found soluble in DMF and DMSO solvents and characterized by using the modern analytical and spectral techniques such as elemental analysis, conductivity, magnetic moments, IR, NMR, UV-visible, Mass, CV, and EPR. The elemental analysis data of ligand and their complexes were well agreed with their calculated values in which metal and ligand stoichiometry ratio 1 : 2 was noted. Molar conductance values indicated that all the complexes were found to be nonelectrolytes. All the complexes showed octahedral geometry around the central metal ions. Herein, we better characterized DNA binding with the complexes by UV-visible and CD spectroscopy and cyclic voltammetry techniques. The DNA cleavage experiments were carried out by Agarose gel electrophoresis method and the cytotoxicity experiments by MTT assay method. Based on the DNA binding, cleavage, and cytotoxicity studies, Cu and Ni complexes were found to be good anticancer agents against AGS-human gastric cancer cell line.

## 1. Introduction

Isatin(1-H indole-2,3-dione) Schiff bases are significant in therapeutic and pharmaceutical compounds in the field [1]. These complexes also exhibited their wide antibacterial [2], antifungal [3], and antitumor activity [4, 5]. Nitrogen containing heterocyclic compounds are identified as indispensable structural units for both the chemists and biochemists [6, 7]. Among the various classes of benzene fused five-membered nitrogen containing heterocyclic compounds, isatin derivatives could be used pharmacologically as an important class of active components [8–10]. The interactions of Schiff base metal complexes containing O and N coordination with DNA have been thoroughly considered [11, 12]. Recently, there has been remarkable interest in studies related to the interaction of transition metal ions with nucleic acid because of their relevance in the development of new reagents for biotechnology and medicine [13]. These studies are also essential to understand the toxicity of drugs containing metal ions [14]. Transition metal complexes have gained significance for their applicability in the biological field [15, 16]. Some of the transition metal complexes, such as Cu(II), were known to function as specific probe for DNA bulges due its ability to cleave DNA [17]. Recently, we have reported our results on the interaction of bidentate Schiff base complexes with DNA [18, 19]. In this background, this study highlights the binding, cleavage, and cytotoxicity with the new series of transition metals N-phenylisatin-isonicotinohydrazide Schiff base complexes with calf thymus DNA and AGS cell line, respectively.

## 2. Experimental

### 2.1. Materials

All chemicals were purchased from Sigma-Aldrich, Merck-(A.R) and used as received without further purification. The isatin Schiff base was prepared according to the literature procedure [20]. N-phenylisatin, isonicotinohydrazide, and DMSO are GR grade; CT and* pUC*-*19 *DNA were purchased from Genie, Bangalore. Metal chlorides [CuCl_2_·2H_2_O, NiCl_2_·6H_2_O, CoCl_2_·6H_2_O] and solvents were purchased from E-Merck, A.R grade, Mumbai.

### 2.2. Physical Measurements

C, H, and N analyses of free Schiff base ligands and their metal complexes were performed in C, H, and N analyzer Elementar Vario EL III. Metal contents were analyzed by the standard procedures. Hand-Held Meter LF330 was used to measure the molar conductance of free Schiff base ligands and metal complexes in DMSO (1 × 10^−3^ M). The electronic spectra were recorded in DMSO solutions using Shimatzu Model 160 UV-visible spectrophotometer. The IR spectra of the complexes were recorded on a JASCO V-550 UV-Vis spectrophotometer in KBr pellets. NMR spectra were recorded on BRUKER DPX-300 High performance Digital FT-NMR spectrometer in DMSO-d^6^ using TMS as internal standard. Electrospray ionisation mass spectrometry (ESI-MS) analysis was performed in the positive ion mode on a liquid chromatography-ion trap mass spectrometer (LCQ Fleet), Thermo Fisher Instruments Limited, US. Magnetic susceptibility measurement of the powdered samples was carried out by the Gouy balance. EPR measurements were carried out by using a Varian E4 X-band spectrometer equipped with 100 Hz modulation. Cyclic Voltammetric measurements were carried out in a Bio-Analytical System (BAS) model CV-50W electrochemical analyzer.

### 2.3. DNA Binding and Cleavage

#### 2.3.1. Electronic Absorption Studies

DNA-binding experiments were performed by UV-visible spectroscopy in Tris-HCl/NaCl buffer (5 mmol L^−1^ Tris–HCl/50 mmol L^−1^ NaCl buffer, pH 7.2) and used DMSO (10%) solution of metal complexes. The concentration of CT-DNA was determined from the absorption intensity at 260 nm with a value of 6600 (mol L^−1^)^−1^ cm^−1^. Absorption titration experiments were made using different concentrations of CT-DNA, while keeping the complex concentration constant. Correction was made for the absorbance of the CT-DNA itself. Samples were equilibrated before recording each spectrum. For metal complexes, the intrinsic binding constant (*K*_*b*_) was determined from the spectral titration data using the following equation [21]:

(1) $$\begin{array}{c} \frac{\left[\text{DNA}\right]}{\left({ℇ}_{\text{a}}-{ℇ}_{\text{f}}\right)}=\frac{\left[\text{DNA}\right]}{\left({ℇ}_{\text{b}}-{ℇ}_{\text{f}}\right)}+\frac{1}{K_{b}\left({ℇ}_{\text{b}}-ℇ{}_{\text{f}}\right)}, \end{array}$$

where *ℇ*_a_, *ℇ*_b_, and *ℇ*_f_ are the molar extinction coefficients of the free complexes in solution, complex in the fully bound from with CT-DNA, and complex bound to DNA at a definite concentration, respectively. In the plot of [DNA]/(*ℇ*_a_ − *ℇ*_f_) versus [DNA], *K*_*b*_ was calculated.

#### 2.3.2. Circular Dichroism (Cd) Measurements

Circular dichroism spectra were registered in a JASCO J-810 spectropolarimeter, using a quartz cuvette of 0.2 cm path length, at room temperature, in the range 230–330 nm. The initial experimental DNA concentration was 800 *μ*M, and the spectra were registered in the absence or in the presence of 10 to 50 *μ*M of each complex studied [22].

#### 2.3.3. Electrochemical Studies

Cyclic voltammetry analysis was carried out in a Bio-Analytical System (BAS) model CV-50W electrochemical analyzer. All voltammetric experiments were performed in a single compartment cell of volume 10–15 mL containing a three electrode system comprising a carbon working electrode, Pt-wire as auxiliary electrode, and reference electrode as an Ag/AgCl.

#### 2.3.4. DNA Cleavage Studies

pUC19 DNA at pH 7.5 in Tris-HCL buffered solution was used to perform Agarose gel electrophoresis. Oxidative cleavage of DNA was examined by keeping the concentration of the 30 *μ*M of complexes and 2 *μ*L of pUC19 DNA and this made up the volume to 16 *μ*L with 5 mM Tris-HCl/5 mM NaCl buffer solution. The resulting solutions were incubated at 37°C for 2 h and electrophoresed for 2 h at 50 V in Tris-acetate-EDTA (TAE) buffer using 1% Agarose gel containing 1.0 *μ*g/mL ethidium bromide and photographed under UV light [23].

### 2.4. Cytotoxicity

Cytotoxicity studies were carried out using human gastric cancer cell line (designated AGS) which were obtained from National Centre for Cell Science (NCCS), Pune, India. Cell viability was carried out using the MTT assay method. The AGS cells were grown in Dulbecco's Modified Eagle's Medium (DME) and Ham's F-12 Nutrient Mixture containing 10% fetal bovine serum (FBS), 1% Glutamine, 1% antibiotic, 1% sodium bicarbonate, and 1% nonessential amino acids. For screening experiment, AGS cells were seeded into 96-well plates in 100 *μ*L of respective medium containing 10% FBS, at plating density of 10,000 cells/well and incubated at 37°C, 5% Co_2_ for 24 h prior to addition of complexes. The complexes were dissolved in DMSO and diluted in the medium. After 24 h, the medium was replaced with respective medium containing the complexes at various concentrations and incubated at 37°C, 5% Co_2_ for 48 h. Triplicate was maintained. After 48 h, 10 *μ*L of MTT (5 mg/mL) in phosphate buffered saline (PBS) was added to each well and incubated at 37°C for 4 h. The medium with MTT was then flicked off and the formed formazan crystals were dissolved in 100 *μ*L of DMSO and then measured the absorbance at 570 nm using microplate reader. The percentage of cell inhibition was determined using the following formula and chart was plotted between percentage of cell inhibition and concentration, and from this IC_50_ value was calculated. percentage of inhibition = [mean OD of untreated cells (control)/mean OD of treated cells (control)] × 100 [24].

### 2.5. Chemistry of the Synthesis Compounds

#### 2.5.1. Synthesis of (*E*)-N′-(2-Oxo-1-phenylindolin-3-lidene) Isonicotinohydrazide (L)

**Scheme 1:**
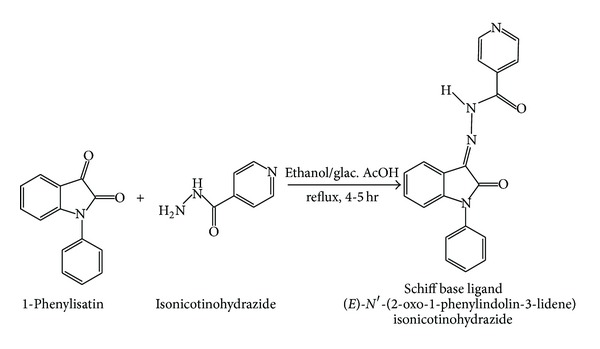
Synthesis of Schiff base ligand.

1-Phenyl isatin (1 mMol) and isonicotinohydrazide (1 mMol) were dissolved in 50 mL of absolute ethanol; three drops of glacial acetic acid were added and the resulting solution was refluxed for 5 h. The results compounds were precipitated upon cooling to room temperature, isolated by filtration, and recrystallized from EtOH. Yellow colored crystalline compounds were obtained (Scheme 1). These ligands were confirmed by Elemental analyzer, IR, NMR, and Mass spectra. Yield: 95%, m.p. 180°C, elemental analysis: found (calculated) (%) for L: C, 70.01 (70.14); H, 3.97 (4.12); N, 16.52 (16.37). IR (cm^−1^ in KBr pellets): 1602 (C=N), 1695 (indole–C=O), 1685 (C=O, isoniazid), 3269 (NH). ^1^H NMR (300 MHz, CDCl_3_, *δ*/ppm): *δ* 14.21 (s, 1H), *δ* 8.80–8.78 (d, pyridine protons, 4H), 7.93–6.86 (m, aromatic protons); ^13^C-NMR 162.15 (C=O, isatin), 161.81 (C=O, isoniazid), 134.55 (C=N, azomethine carbon), 150.91–110.12 (aromatic ring) ESI-MS = 343 (M+H).

#### 2.5.2. Synthesis of Cu(II), Co(II), and Ni(II) Complexes

**Scheme 2:**
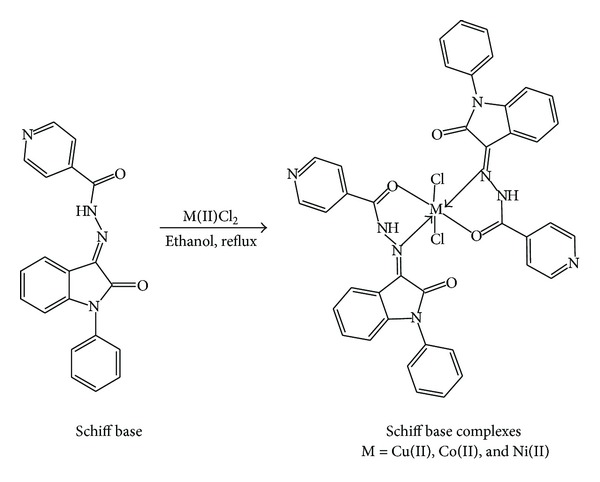
Synthesis of Schiff base complexes.

The metal(II) complexes in this study were prepared by mixing of 1 mMol of corresponding metal(II) chloride in ethanol with 2 mM of the Schiff base in the molar ratio 1 : 2. The reaction mixture was refluxed at 60°C for 4 hrs [25]. Then it was allowed to cool at room temperature. Powdered solid obtained was filtered, washed with ethanol, and dried under vacuum (Scheme 2). The Schiff base complexes were characterized by elemental analysis, UV-visible, infrared (IR), electron paramagnetic resonance (EPR) spectroscopy, and magnetic moment.

*Complex 1.* Yield: 83%, m.p. > 300°C, elemental analysis: found (calculated) (%) for L–Cu: C, 59.37 (59.03); H, 3.71 (3.75); N, 13.11 (13.43). UV-visible (in MeOH): *λ*_max⁡_ (nm): 278(ILCT), 344(ILCT), 454(MLCT), 810 (d–d). IR(cm^−1^): 1612 (C=N), 1691 (indole–C=O), 1673 (–NH–C=O), 3265 (NH), 601 (M–O), 453(M–N), *g*_||_ = 2.339; *g*_⊥_ = 2.05; A_||_ = 120 × 10^4^, *μ*_eff_ (300 K): 2.95*μ*_B_.

*Complex *2. Yield: 80%. m.p. > 285°C elemental analysis: found (calculated) (%) for L–Co: C, 59.32 (59.36); H, 3.63 (3.77); N, 13.47 (13.51). UV-visible (in DMSO): *λ*_max⁡_ (nm): 276(ILCT), 344(ILCT), 612, 674 (d–d). IR (cm^−1^): 1612 (C=N), 1693 (indole–C=O), 1676(–NH–C=O), 3263(NH), 572(M–O), 447 (M–N), *μ*_eff_ (300 K): 4.52 *μ*_B_.

*Complex 3.* Yield: 80%. m.p. > 285°C, elemental analysis: found (calculated) (%) for L–Ni: C, 59.35(59.38); H, 3.68 (3.77); N, 13.56 (13.51). UV-visible (in DMSO): *λ*_max⁡_ (nm): 274(ILCT), 344(ILCT), 452(MLCT). IR (cm^−1^): 1610(C=N), 1693(indole–C=O), 1674 (–NH–C=O), 3268 (NH), 574 (M–O), 449 (M–N), *μ*_eff_ (300 K): 3.41 *μ*_B_.

## 3. Results and Discussion

The Schiff base ligand (L) and their complexes with Cu(II), Co(II), and Ni(II) were found to be air stable, amorphous, moisture free, and soluble only in DMF and DMSO solvents and kept in vacuum desiccators under nitrogen atmosphere and used for chemical and biological studies. The experimental results are discussed under various subheadings as detailed below.

### 3.1. Elemental Analysis and Conductivity Measurements

Physicochemical characteristics such as melting point (m.p.), color, yield, elemental analysis, and conductivity of the ligand(L) and complexes were determined and the data shown in Table 1. The observed low conductivity values (22.0–38.40 *Ω* ^−1^ cm^2^ mol^−1^) were accounted for the dissociation and hence the complexes are found as nonelectrolytes [26].

### 3.2. NMR Spectra

**Figure 1:**
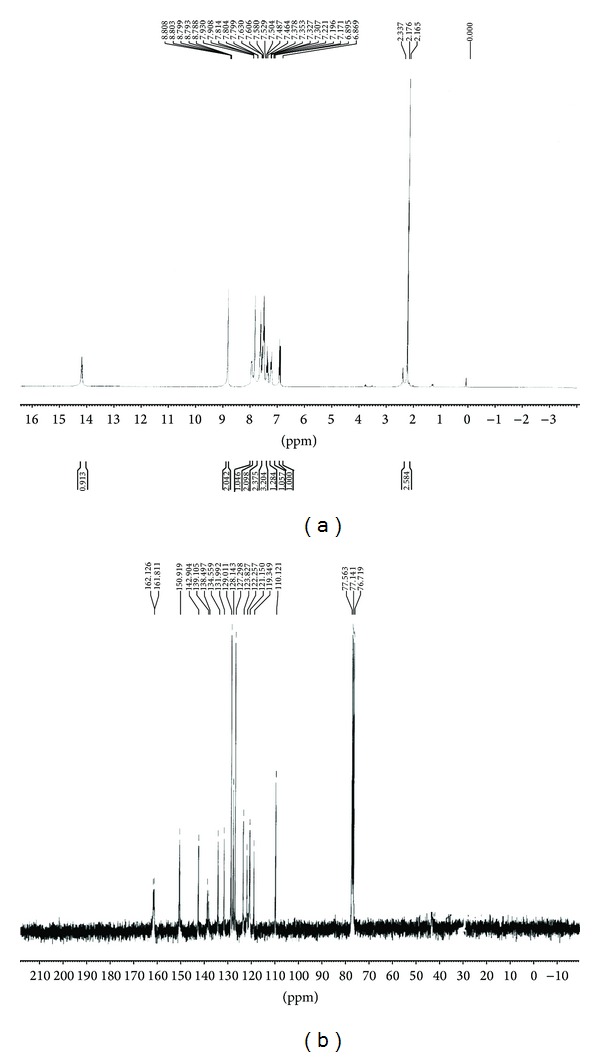
(a) ^1^H NMR spectrum and (b) ^13^C NMR of ligand.

The ^1^H-NMR (300 MHz, CDCl_3_, *δ*/ppm) spectrum of the Schiff base which exhibited a signal at 14.21(s, 1H) was assigned to the NH proton of isonicotinohydrazide and the signals at 7.93–6.86 (m, 13H) were assigned to aromatic protons (Figure 1(a)). The ^13^C NMR spectra provide further support for the structure evidence of the ligand. The signals at 162.15 and 161.81 confirm the carbonyl carbon of isatin and isonicotinohydrazide. The signals appeared at 134.55; it confirms the formation of imine carbon and signals from 150.91 to 110.12, the aromatic rings (Figure 1(b)).

### 3.3. Analysis of Mass Spectra

**Figure 2:**
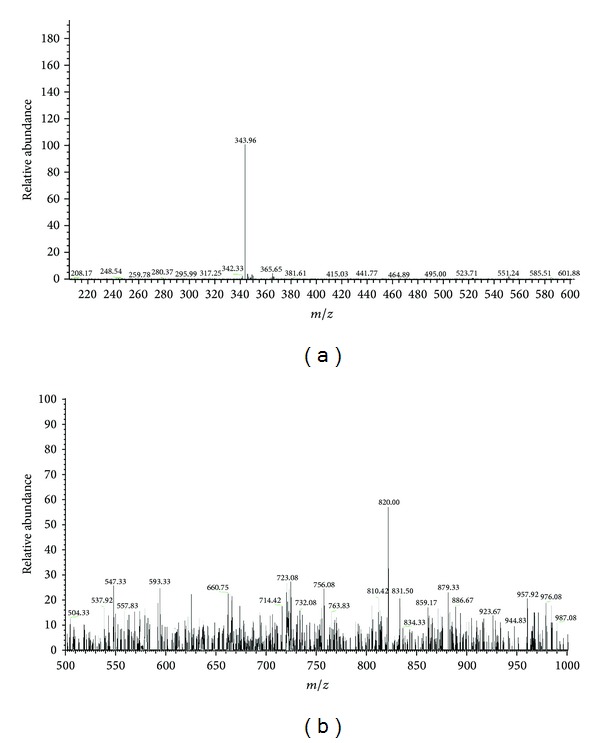
Mass spectrum of ligand (a) and copper complex (b).

Mass spectrometry (MS) an analytical technique that measures the mass-to-charge ratio of charged particles. ESI mass spectra for ligand and complexes were recorded and are shown in Figure 2. MS[ESI (M+1)] exact mass calculated for L (a) required* m/z* 342.81 and found* m/z* 343.96 and copper(II) (b) complex required* m/z* 819.9 and found* m/z* 820. These values also confirm the formation of ligand and complexes.

### 3.4. Infrared Spectra

In order to study the bonding mode of ligand moiety to metal ion in the complexes, IR spectra of the free ligand were compared with those of the metal complexes. The FT-IR spectral data are summarized in Table 2. The IR spectrum of the free ligand (L) showed broadband at 3269 cm^−1^, which can be attributed to NH stretching vibration of the isoniazid structural unit. The position of these bands remained at nearly the same frequency in the spectra of the metal complexes which suggests the noncoordination of this group to central metal ion in the metal complexes [27]. A sharp peak at 1602 cm^−1^ was assigned to *ν*(C=N), which is characteristic of Schiff bases. In the spectra of the complexes, this peak is slightly shifted to higher frequency around 1610–1612 cm^−1^. This suggested that one point of attachment of the metal is through the azomethine nitrogen atom [28, 29]. The strong intensity bands of ligand were observed at the region 1685 cm^−1^ of the spectra indicating carbonyl group. The position of these bands was shifted to lower region 1673−1676 cm^−1^, indicating the involvement of *ν*(C=O) with metal centre during complexation. The bands at 1695 cm^−1^ and 1691–1693 cm^−1^ in the spectrum of the free ligand and complexes, respectively, were assigned to *ν*(C=O) of isatin moiety. The positions of these bands were found at nearly the same frequency in spectra of the metal complexes, suggesting the uncoordination of this group. New bands observed in the 447–453 and 572–601 cm^−1^ for the complexes were assigned to stretching frequencies of M–N and M–O, respectively [30, 31]. Thus, the IR spectral results provide evidence for bidentate complex formation of Schiff bases with metals.

### 3.5. Electronic Spectra and Magnetic Moment Values

The electronic spectra of the ligand and its Cu(II), Co(II), and Ni(II) complexes were recorded in DMSO and their probable assignments are given in Table 3. The absorption bands at 36764 cm^−1^ and 29239 cm^−1^ attributed to *π* → π* and *n* → π* transitions in the ligand(L). The Cu(II) complex showed d–d band at 12345 cm^−1^. These bands may be assigned to ^2^B_1g_ → ^2^B_2g_, ^2^E_g_ transitions. The position of these bands is consistent with octahedral geometry around the Cu(II) ion. The electronic spectra of Co(II) complex exhibited the absorption d–d bands at 16339 and 14836 cm^−1^. These bands may be assigned to ^4^T_1g_(F) → ^4^T_1g_(P) and ^4^T_1g_(F) → ^4^A_2g_ transitions. The position of these bands is consistent with octahedral geometry around the Co(II) ion. The Cu(II) and Co(II) complexes showed paramagnetism, 4.52 and 4.13 BM respectively [32, 33]. Similarly, the electronic spectra of Ni(II) complex exhibited absorption band at 22132 cm^−1^ and assigned to be LMCT band and the d–d band suppressed by LMCT band. The position of these bands is consistent with octahedral geometry around the Ni(II) ion. The Ni(II) also showed paramagnetism, 3.91 BM [34].

### 3.6. EPR Spectra

**Figure 3:**
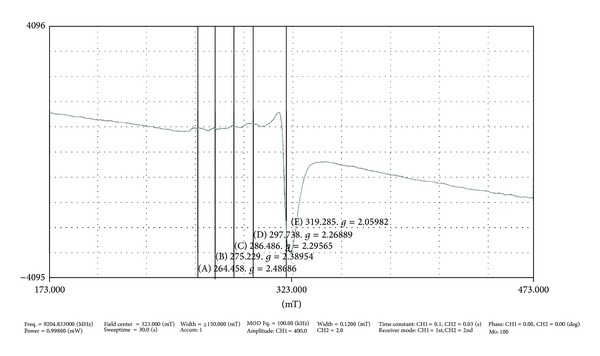
EPR spectrum of copper complex at 77 K.

The X-band EPR spectrum of the copper(II) complex was recorded in the solid state at room temperature and in DMSO solvents at liquid nitrogen temperature using the DPPH radical as the *g* marker (Figure 3). The complex has a well-resolved *g*_||_ and broadened *g* regions and various Hamiltonian parameters have been calculated as *g*_||_ = 2.339; *g*_⊥_ = 2.05; *A*_||_ = 120 × 10^4^. The trend *g*_||_ > *g*_⊥_ observed in this complex indicates that the unpaired electron is most likely to be in the *d*_*x*^2^–*y*^2^_ orbital [35].

### 3.7. Cyclic Voltammetry

**Figure 4:**
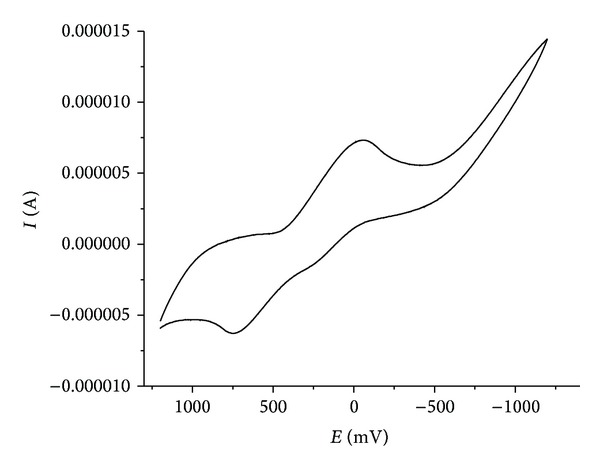
CV spectrum of Cu(II) complex.

A cyclic voltammogram of Cu(II) complex presented in Table 4. Voltammogram (Figure 4) displays a reduction peak at Epc = −103.60 mV, with an associated oxidation peak at Epa = −214.77 mV at a scan rate of 50 mV/s. The peak separation of this couple (ΔEp) is 0.77V and increases with scan rate, I_pa_/I_pc_ = 1.03. Thus, the analyses of cyclic voltammetric responses at different scan rate gave the evidence for quasireversible one electron reduction. The most significant feature of the Cu(II) complex is the Cu(II)/Cu(I) couple. The ratio of cathodic-to-anodic peak height was less than one. However, the peak current increases with the increase of the square root of the scan rates. This establishes the electrode process as diffusion controlled [29]. In Co(II) complex shows a redox process corresponding to the Co(II)/Co(I) couple at Epa = 790 mV and the associated cathodic peak at Epc = 610 mV and the Ni(II) complex showed redox process corresponding to the Ni(II)/Ni(I) couple at Epa = 521 mV and the associated cathodic peak at Epc = 384 mV. These couples are also found to be quasi-reversible as the peak separation between the anodic and cathodic potentials. But the ratio between the anodic and cathodic currents suggests that the process is simple one-electron transfer, quasi-reversible process [36].

### 3.8. DNA Interaction Studies

DNA is a molecule of great biological significance and controls the structure and function of cells [36]. These important biological activities will be started via receiving a signal to DNA, which is often in the form of a regulatory protein binding to a particular region of the DNA molecule. The binding specificity and strength of this regulatory protein may be imitated by a small molecule; consequently DNA function can be artificially modulated, inhibited, or activated by binding this molecule instead of the protein [37]. Some studies show that binding can occur between the DNA base pairs (intercalation) [38, 39], while some results are indicative of their groove binding nature [40, 41]. DNA interaction studies have been carried out with the prepared complexes by using UV-visible, CD, and CV spectral techniques and DNA cleavage activity also has been studied by gel-electrophoresis which showed significant results.

#### 3.8.1. DNA Binding-Absorption Spectra

The change of the UV spectra of complexes in the presence of different concentrations of DNA was studied. Hypochromism and red shift in the UV absorption spectra were observed upon addition of DNA increasing concentrations to the complexes solution in the absorption intensity region 275–280 nm and 344–346 nm. These effects are particularly pronounced for intercalators. In the case of groove binders wavelength shift is usually correlated with a conformational change on binding or complex formation [42]. In general, the extent of the hypochromism indicates the interaction binding strength and intrinsic binding constant, *K*_*b*_ for complexes with CT-DNA was determined according to (1) [43], where [DNA] is the concentration of DNA in base pairs. *ℇ*_a_ is the extinction coefficient for APM absorption band at a given DNA concentration, *ℇ*_f_ is extinction coefficient of free complexes, and *ℇ*_b_ is the extinction coefficient of complexes when fully bound to DNA (it is assumed when further addition of DNA does not change the absorbance).

**Figure 5:**
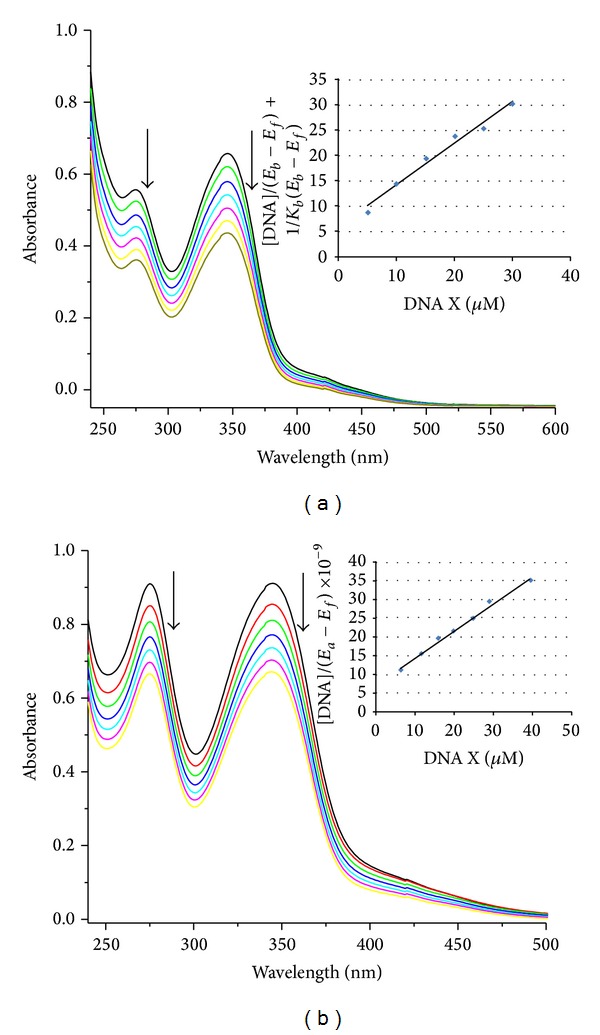
Absorption spectra of (a) Cu(II) and (b) Co(II) complexes in the absence and in the presence of the CT-DNA. [complex] = 30 *μ*M, [DNA] = 0 to 30 *μ*M. The arrow indicates absorption intensity decrease with increasing addition of the CT-DNA. Plots of [DNA]/(*ℇ*_a_ − *ℇ*_f_) versus [DNA] for the complexes with CT-DNA. The arrow indicates absorption intensity decrease with increasing addition of the CT-DNA.

In particular, *ℇ*_f_ was determined by a calibration curve of the isolated complexes in DMSO solution, following Beer's law. *ℇ*_a_ was determined as the ratio between the measured absorbance and the complex concentration. Plot of [DNA]/(*ℇ*_a_ − *ℇ*_f_) versus [DNA] gives a slope of 1/(*ℇ*_b_ − *ℇ*_f_) and a *y*-intercept equal to 1/*K*_*b*_(*ℇ*_b_ − *ℇ*_f_); *K*_*b*_ is the ratio of the slope of the *y*-intercept (Figure 5 insert). The *K*_*b*_ value was calculated to be 10.50 × 10^4^, 5.88 × 10^4^, and 6.81 × 10^4^ M^−1^ (Table 5). The *K*_*b*_ value obtained here is less than that of reported for classical intercalator (for ethidium bromide whose binding constants have been found to be in the order of 10^6^–10^7^ M^−1^) [44]. In comparing the intrinsic binding constant (*K*_*b*_) of Cu(II), Co(II), and Ni(II) complexes with DNA groove binders, as observed in the literature, we can deduce that this complex binds to CT-DNA via groove binding [45, 46].

#### 3.8.2. DNA Binding-Cd Spectra

**Figure 6:**
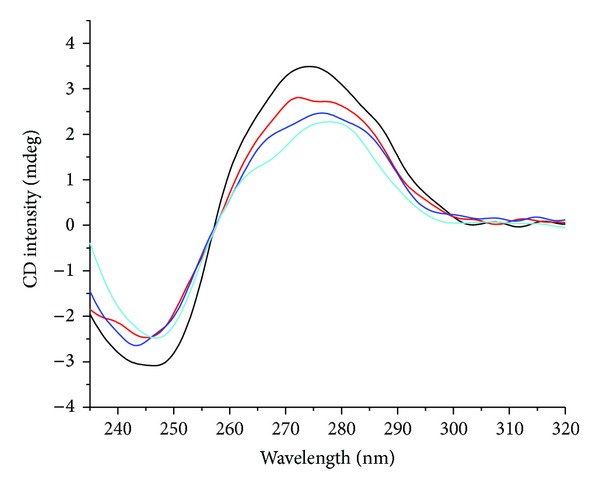
Circular dichroism spectra of DNA (80 *μ*M) in 10 mM Tris HCl buffer, in the presence of increasing amounts of copper(II) complex.

CD spectra is a useful technique in diagnosing changes in DNA morphology during drug–DNA interactions, since CD signals are quite sensitive to the mode of DNA interactions with small molecules [47]. In the case of CT-DNA interacting with metal complexes, the characteristic CD spectra showed two bands as a positive one at 275 nm due to the base stacking between the compounds and DNA bases and a negative band at 245 nm due to the right-handed helicity B form of DNA [48]. Observed changes in these CD signals of DNA are usually assigned to corresponding changes in its structure (Figure 6). The simple groove binding or electrostatic interaction between small molecules and DNA causes less or no perturbation on the base stacking and helicity bands, whereas a classical intercalation enhances both CD bands, stabilizing the CT-DNA form B conformation, as observed for intercalative ligands [49]. Complexes Cu, Co, and Ni exhibited different binding constant values, determined in UV-visible experiments. Generally the DNA of A and B forms structures which have right-handed helix; however, the helical parameters are different in helix pitch, base pair tilt, and twist angle in degrees as 28, 20, and 33 (A form) and 24, −6, and 36 (B form), respectively. B form is the major form that is found in the cell (Watson and Crick 1953). However, after the complex addition to CT-DNA it was only verified as small perturbations in negative and positive bands of CD spectra for three complexes, as shown in Figure 6. Macías et al. [50] observed an increase in both positive and negative bands after incubating complexes with DNA, attributed to a typical intercalative mode, involving *π* → π* stacking and stabilization of the right-handed form of CT-DNA. Incubation of DNA with complexes shows little perturbation of the two bands, which is indicative of a nonintercalative interaction between complexes and DNA and offers another support to its groove binding nature [51].

#### 3.8.3. DNA-Binding-Cyclic Voltammetry Study

**Figure 7:**
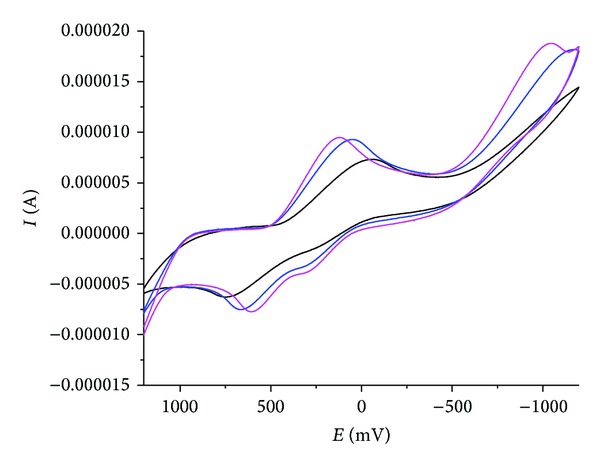
Cyclic voltammogram of copper(II) complex in the absence and presence of increasing amounts CT-DNA at room temperature in DMSO: buffer (1 : 2) mixture (pH 7.2) (scan rate 0.1 Vs^−1^).

Electrochemical investigations of metal-DNA interactions provide a useful complement to spectroscopic methods. Cyclic voltammogram of copper complex in the presence of CT-DNA in various concentrations is shown in Figure 7. CV data explored that Cu exhibited a pair of redox peaks for one electron transfer couple of Cu(II)/Cu(I) at the scan rate of 50 mVs (curve). The ratio of (Ipa/Ipc) value of 0.5 and the peak to peak separation (ΔEp) of 0.44 V suggested the characteristic of the of the electrotransfer process and this was fairly common for Cu(II)/Cu(I) couple because of the reorganization of the coordination sphere. After interaction with CT-DNA, the value of ΔEp was decreased to 0.23 V suggesting that the reversibility of the electron-transfer process of the copper complex was changed better. Moreover, both the oxidation and the reduction peak potentials underwent positive shifts accompanied by the decreases of the redox peak currents. It has pointed out that the electrochemical potential of the small molecules would shift positively when it interacted into DNA double helix, and if it bounds to DNA by groove binding only takes place. So we thought that the greater affinity of Cu with CT-DNA is most likely caused by a specific binding mode [52]. The two quasireversible redox couple for Cu(II) complex and other complexes are irreversible redox couple for Co(II) and Ni(II).

#### 3.8.4. DNA Cleavage Studies By Agarose Gel Electrophoresis

**Figure 8:**
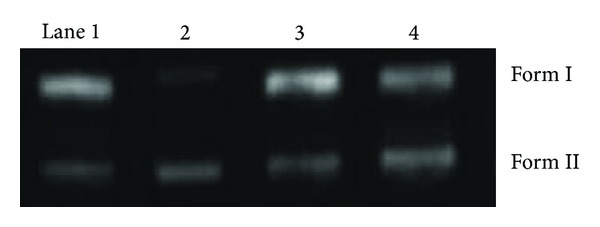
Cleavage of super coiled pUC19 (10 *μ*M) by the Cu(II), Co(II), and Ni(II) complexes in the presence of triacetate EDTA (TEA) buffer at 37°C. Upper line: Form I and lower line: Form II; Lane 1: DNA-control; Lane 2: L–Cu; Lane 3: L–Co; Lane 4: L–Ni.

The ability of Cu(II), Co(II), and Ni(II) complexes to perform DNA cleavage was monitored by agarose gel electrophoresis with the pUC19 plasmid DNA. The experimental results were shown in Figure 8. Two clear bands were observed for the controls in which the three complexes were absent (lane 1). The relatively fast migration is the intact super coil form (Form II) and the slower moving migration is the open circular form (Form I), which was generated from super coiled when scission occurred on its one strand [53, 54]. The amount of Form II diminished gradually and partly converted to Form I and it is obvious that the complexes have more ability to cleave the super coiled plasmid DNA.

### 3.9. Cytotoxicity

**Figure 9:**
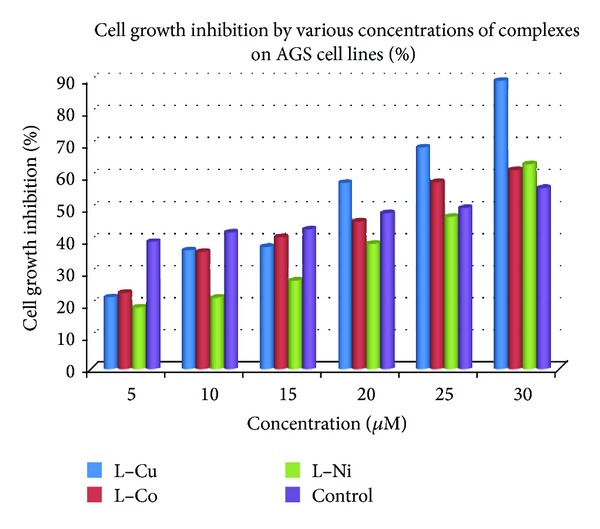
Treatment of complexes that exert an antiproliferative effect on liver cancer cell line. HepG2 cells were treated with complexes (Cu, Co, and Ni) for 48 h. Control received appropriate carriers. Cell viability was assessed by MTT cell proliferation assay.

The cytotoxicity assay for the new complexes was assessed using the method of MTT reduction. The market reference Mitomycin-C was used as a positive control. All the ligands and complexes were found to be cytotoxic to liver cancer cell line (HepG2). The IC_50_ values (50% inhibition of cell growth for 48 h) for complexes Cu, Co, and Ni are 5 *μ*M, 10 *μ*M, 20 *μ*M, 25 *μ*M, and 30 *μ*M, respectively (Figure 9). The complexes exhibited higher cytotoxic effects on liver cancer cells with lower IC_50_ values indicating their efficiency in killing the cancer cells even at low concentrations. The ligand did not show any significant activity up to 100 *μ*M. However, cytotoxic effectiveness of the compounds with an IC_50_ of Cu and Ni complexes was higher than that of control. There are reports in the literature on the cytotoxic effects of the complexes with longer incubation time periods [55–57]. The longer incubation period may result in the development of cellular resistance for that particular complex. The complexes Cu and Ni is showed better activity than Co complex because the reduction potential of Cu(II) and Ni(II), almost in same order of magnitude; however the Co(II) is widely varied and mostly biocompatible in living system. Moreover, the IC_50_ values of our complexes are comparable with the reported IC_50_ values of standard anticancer drugs such as Mitomycin-C.

## 4. Conclusion

One of the most important goals of pharmacological research is the search for new molecular structures which exhibit effective antitumor activities. This has driven inorganic and organometallic chemists to look for new metal compounds with good activities, preferably against tumors that are responsible for high cancer mortality. In this study, new series of Schiff base (L) and its complexes Cu(II), Co(II), and Ni(II) showed octahedral geometry. The binding behaviors of the complexes toward CT-DNA were investigated by absorption spectroscopy, CD, and CV techniques. In conclusion competitive binding of complexes for DNA indicated that complexes could interact as a groove binder. It should be noted that the observed intrinsic binding constant (5.88–10.50 × 10^4^ M^−1^) is comparable to other groove binders as well and complexes Cu and Ni have stronger binding affinity than Co. The complexes bind to super coiled plasmid pUC19 DNA and display efficient hydrolytic cleavage and are a specific groove binder. The cytotoxic studies showed that the complexes Cu and Ni exhibit good cytotoxic activity against AGS cell line. Furthermore, these complexes have potential practical applications to formulate into an efficient drug against cancer.

## Figures and Tables

**Table 1:** Composition and physical characteristics of ligand and their complexes.

Ligand/ complexes	Molecular formula	Color	Found (calculated) %				M.P (°C)	Yield (%)	*Ω* (Ohm^−1^ cm^2^ M^−1^)
			M	C	H	N			
L	C_20_H_14_N_4_O_2_	Crystalline yellow		70.01 (70.14)	3.97 (4.12)	16.52 (16.37)	180	95	—
L–Cu	C_41_H_31_N_8_O_4_Cl_2_Cu	Green	7.51 (7.62)	59.37 (59.03)	3.71 (3.75)	13.11 (13.43)	>300	83	22.000
L–Co	C_41_H_31_N_8_O_4_Cl_2_Co	Dark green	6.58 (7.10)	59.32 (59.36)	3.63 (3.77)	13.47 (13.51)	>285	80	34.40
L–Ni	C_41_H_31_N_8_O_4_Cl_2_Ni	Yellow	7.31 (7.08)	59.35 (59.38)	3.68 (3.77)	13.56 (13.51)	>285	80	28.50

**Table 2:** Infrared spectral data for the free ligand and their complexes in KBr disc (cm^−1^).

Compounds	C=N (imine)	C=O (isatin)	C=O (isoniazid)	NH	M–O	M–N
L	1602	1695	1685	3269	—	—
L–Cu	1612	1691	1673	3265	601	453
L–Co	1612	1693	1676	3263	572	447
L–Ni	1610	1693	1674	3268	574	449

**Table 3:** Electronic spectral parameters and magnetic moment with suggested geometry of the complexes.

Compound	*π*→π* (cm^−1^)	*n*→π* (cm^−1^)	LMCT	d-d band	Assignment	Suggested structure	*μ*_eff_ (B.M)
L	36764	29239					
L–Cu	35971	29069	22026	12345	^ 2^B_1g_→^2^B_2g_, E_g_	Distorted octahedral	4.52
L–Co	36231	29068	—	16339, 14836	^ 4^T_1g_(F)→^4^T_1g_(P)	Octahedral	4.13
L–Ni	36496	29069	22132	—	^ 3^A_2g_→^3^T_1g _(F)	Octahedral	3.91

**Table 4:** Electrochemical parameters for Cu(II), Co(II), and Ni(II) complexes.

Compound	Redox couple	Epa (mV)	Epc (mV)	ΔEp (mV)	Ipa/Ipc
L–Cu	Cu(II)/Cu(I)	−214	−103	111	1.03
L–Co	Co(II)/Co(I)	790	610	180	0.82
L–Ni	Ni(II)/Ni(I)	521	384	137	0.91

**Table 5:** Absorption properties of metal (II) complexes with CT-DNA.

Complexes	*λ*_max⁡_ (nm)	Δ*λ* (nm)	Hypochromicity (%)	*K*_*b*_ (M^−1^)
L–Cu(II)	275, 345	3	59.05, 65.45	10.50
L–Co(II)	280, 346	5	37.53, 42.18	5.88
L–Ni(II)	275, 344	2	37.53, 58.48	6.81
